# Short-Term Alteration of Soil Physicochemical Characteristics Induced by Biochar Application on a *Ferric Acrisol*

**DOI:** 10.1155/sci5/7743251

**Published:** 2025-02-06

**Authors:** Jochebed Kaki Torgbenu, Godfred Boateng, Felix Osei Kwarteng, Stephen Ardey Mensah, Edward Benjamin Sabi, Peace Korshiwor Amoatey, Peter Amoako Ofori, Stella Owusu-Nketia, Anthony Raphael Simpson, Selorm Yaotse Dorvlo, Emmanuel Essien, Evans Asenso

**Affiliations:** ^1^Department of Agricultural Engineering, School of Engineering Sciences, University of Ghana, Accra, Ghana; ^2^Biotechnology Center, College of Basic and Applied Sciences, University of Ghana, Accra, Ghana; ^3^Department of Crop Science, School of Agriculture, University of Ghana, Accra, Ghana

**Keywords:** biochar, C sequestration, carbon-based matter, coconut husk biochar, soil health and quality, sugarcane bagasse biochar

## Abstract

Biochar (incinerated organic waste by-product) has shown promise in enhancing soil fertility and agricultural productivity. Soil quality plays an essential role in the success of agricultural activities, with soil enhancement being crucial for optimizing crop yields and fostering soil fertility. An experiment with different biochar types was arranged in a randomized complete block design. The biochar [coconut husk (CH) biochar and sugarcane bagasse (SB) biochar] was evenly hand mixed with the soil after plowing to 30 cm depth. A one-time application of biochar was done. There was a total of six treatments: SB biochar, SB biochar plus nitrogen (N), phosphorus (P), potassium (K) (SB + NPK), CH biochar, CH biochar plus NPK (CB + NPK), NPK, and control (CT), with three replicates for each treatment. The area of each plot was 3 m^2^ (3 m × 1 m) to assess the effects of biochar application on the soil physical and chemical characteristics of *Ferric Acrisol* with cabbage *(Fortune F1 variety)* as a test crop in Ghana. Soil bulk density, porosity, pH, organic carbon (OC), available N, total P, available K, available calcium (Ca), electrical conductivity (EC), and cation exchange capacity (CEC) were determined. CH and SB addition improved soil bulk density (1.21 g·cm^−3^ and 1.29 g·cm^−3^), leading to a significant (*p* < 0.05) improvement in the total porosity (54.29% and 51.10%), respectively, at 0–30 cm soil depth compared to the presoil condition (1.5 g·cm^−3^). Additionally, CH and SB significantly (*p* < 0.05) impacted the soil chemical characteristics and fertility of the tested soil. The results showed that biochar application is crucial for C sequestration, reduction in pH (SB-7.36 and CH-7.44 compared to the presoil condition (4.93) at 0–30 cm soil depth), and soil fertility enhancement. Applying biochar to soils can therefore be considered a potential solution to improve soil fertility for sustainable crop production.

## 1. Introduction

A decline in crop productivity poses a serious threat to food security in sub-Saharan Africa (SSA) due to various constraints, including soil degradation, adverse climatic conditions, pests, and diseases [1]. The Coastal Savannah Agroecological Zone of Ghana, covering approximately 20,000 km^2^ [2], is predominantly characterized by *Ferric Acrisol* soils. Although these soils are widely cultivated, their primary characteristic is low-activity clay composition [3].

The declining soil fertility status in this region necessitates integrated and sustainable interventions to enhance productivity. Major vegetable crops (including tomato, okra, cabbage, and lettuce) and grain crops such as maize and rice exhibit low yields due to poor soil characteristics. Continuous cultivation results in rapid decline of organic matter content, resulting in reduced levels of essential soil nutrients such as nitrogen and phosphorus [4]. Various soil management systems and traditional farming practices have been suggested to address low nutrient levels. These approaches encompass agroforestry, manuring, cover cropping, mulching, shifting cultivation, and crop rotation [5]. Sustainable soil and crop management practices, such as biochar application, have recently emerged as a promising intervention strategy for soil quality improvement [6].

A by-product of the pyrolysis of organic waste, biochar is a solid, carbonaceous, finely textured material with a high specific surface [7]. Its application has shown a significant potential in enhancing soil fertility and nutrient use efficiency, both of which play crucial roles in promoting crop production [3, 7] as well as mitigating climate change through carbon sequestration [8]. In addition, biochar application leads to notable improvements in various aspects of soil health and quality, including biological properties (soil microbial activities and interactions) and physical properties like water-holding capacity, and bulk density, as well as chemical properties such as pH and cation exchange capacity (CEC) [9–11]. It is an economical solution for enhancing soil fertility in agricultural lands [12] and a sustainable waste management or renewable resources recycling measure to protect the environment [13]. However, the impact of biochar on the content and composition of dissolved organic matter (DOM) in soils has been a relatively understudied area [8].

In Africa, the increase in the production of agricultural waste could be beneficial by generating more biochar, but this waste is currently mismanaged through direct disposal and open burning, leading to severe environmental pollution and its related consequences [14]. Annually, Ghana alone produces approximately 3 million tons of organic waste [15] including sugarcane bagasse (SB), rice husks, coconut husks (CHs), coffee husks, groundnut husks, oil palm fruit bunch, plantain and banana peduncle, and various residues from forest-related activities such as wood shavings, bark, chips, and sawdust. Addressing this challenge necessitates sustainable waste management strategies, particularly to achieve Sustainable Development Goal (SDG) 6 for improved sanitation.

In Ghana, there is a sustainable approach to handling coconut biomass waste involving the production of cocopeat for greenhouse vegetables and cocoa seedling cultivation. Nevertheless, there is a need to explore alternative methods of managing CH and SB that can contribute to the production of biochar to enhance soil health and quality. The objective of this study was to explore the utilization of biochar produced from CH and SB for improving *Ferric Acrisol* soil for vegetable production.

## 2. Materials and Methods

### 2.1. Description of the Site and Experimental Materials

**Figure 1:**
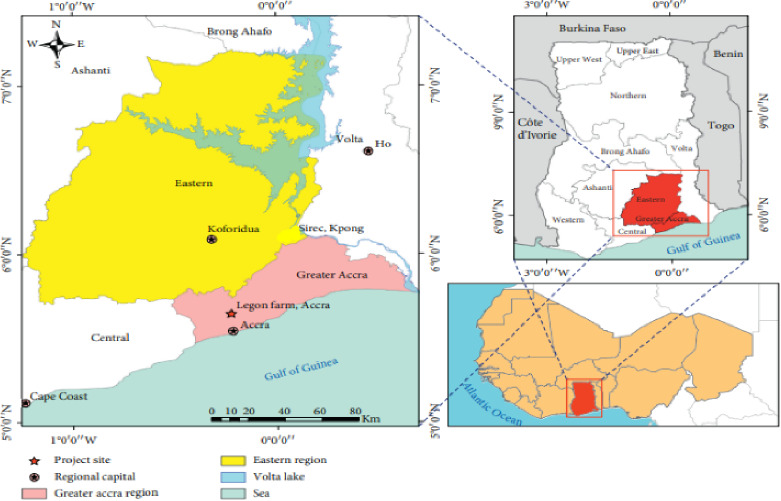
Site location of the University of Ghana Research Station, Legon, Accra, Ghana.

The experiment was conducted at the University of Ghana Research Station, Legon, Accra Ghana, during the 2022/2023 growing season. The Research Station is located at 5°70′20.4 N and 0°19′69.4 W (Figure 1). The soil of the experimental site is mainly *Ferric Acrisol*. The Ferric are coarser in texture than Acrisol and their textural classes that range between sand and silt loams. The soil is brown in color and turns to dark-brown when moist. Generally, the wide-spread occurrence of salinity and sodicity problem is mainly due to the weathering of Na-, Ca-, Mg-, and K-rich igneous rocks. The climatic condition for the experimental site is shown in Table 1.

### 2.2. Experimental Design

The experimental plots were arranged in a randomized complete block design. The biochar was evenly hand mixed with the soil after a 0–30 cm depth of the plow layer. A level of biochar at 100% was applied. There were a total of six treatments: SB biochar, SB biochar plus NPK (SB + NPK), CH biochar, CH biochar plus NPK (CB + NPK), NPK, fertilizer only and control (CT), and three replicates adopted for each treatment. The area of each plot was 3 m^2^ (3 m × 1 m). Before transplanting the cabbage seedlings (variety *Fortune F1*), each plot was separated by a waterproof polyethene–polypropylene fabric that was buried vertically at 1 m and did not disturb the soil in the plot. Subsequently, the biochar (5.0 kg) was hand applied uniformly to each plot. The spacing of the cabbage was 0.70 m between row and 0.50 m within row. 2.0, 5.0, and 5.0 g of NPK were applied on the third day after transplanting, the second week, and the fifth week, respectively, to the biochar's and NPK combined, as well as the sole or lone treatment. Weeds and insects were controlled manually, by handpicking and spraying of insecticides, respectively.

### 2.3. Soil Characterization

#### 2.3.1. Soil Physical Characterization

Selected soil physical characteristics including bulk density, porosity, and soil texture were determined prior to the start of the experiment. Soil samples were collected randomly from five different points in the field at 0–10, 10–20, and 20–30 cm soil depth using a core sampler with 5-cm-diameter core rings to determine initial soil bulk density and porosity (Table 2). However, soil samples were also randomly collected from three points in each individual plot at 0–10, 10–20, and 20–30 cm soil depths after harvesting to determine the bulk density and porosity. The samples were taken to the laboratory and oven-dried at 105°C for 24 h. The soil bulk density was then determined according to the method as described by Blake [16]. The total porosity was calculated from particle density and bulk density using the following equation:

(1) $$\begin{array}{c} \text{total porosity}=\left(1-\frac{\rho_{b}}{\rho_{s}}\right), \end{array}$$

where *ρ*_*b*_ is the bulk density and *ρ*_*s*_ is the particle density, assuming a particle density of 2.65 g·cm^−3^ [17]. Soil texture was determined using the hydrometer method as described by Bouyoucos [18].

#### 2.3.2. Soil Chemical Characterization

The following selected soil chemical characteristics, pH, organic C, available N, available K, Ca, total P, EC, and CEC as shown in Table 2, were determined prior to and after harvesting. Soil samples were randomly collected from five points prior from the whole field and three points in each individual plot at 0–10, 10–20, and 20–30 cm soil depths, respectively, using a soil auger. Soil pH was determined using a pH meter in a 1:2.5 soil:water ratio as adopted by Bremmer and Mulvaney [19]. CEC and exchangeable cation were determined by ammonium acetated extraction method [19]. Available N was determined by the Kjeldahl method [20]. Total K was determined by the ammonium acetate extraction method [19]. Organic C was determined by the Walkley and Black method [21].

### 2.4. Biochar Characterization

**Figure 2:**
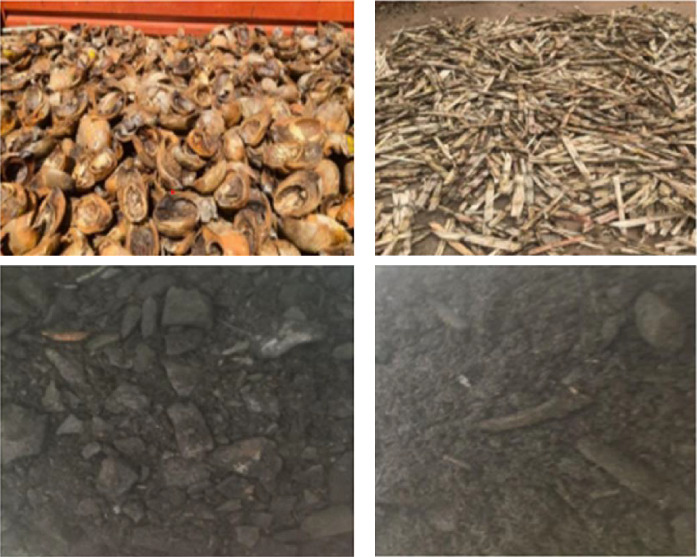
(a): Coconut husk (CH) biochar and (b): sugarcane bagasse (SB) biochar.

The biochar raw material was made up of CH (Figure 2(a)) heated at 500°C [22] and SB(Figure 2(b)) at 210°C [23] under anaerobic conditions, and the retention time of the pyrolysis process was 1 h. The biochar was analyzed for the following chemical characteristics: pH, organic C, available N, total P, available K, and EC (Table 3), using the standard methods for the determination of soil chemical characteristics.

### 2.5. Statistical Analysis

Statistical analysis and graphs were plotted with IBM SPSS 23.0 (SPSS Inc. Chicago, USA). Each data point was summarized by calculating the average value, and the analysis of variance and mean comparisons (Duncan's multiple range test, *p* < 0.05) were conducted. Pearson's correlation, the dendrogram of ward linkage from the hierarchical cluster, and biplot analysis were carried out to reveal the relationships among the physical and chemical characteristics of biochar on the soil.

## 3. Results and Discussion

### 3.1. Soil and Biochar Characteristics

The CH- and SB-derived biochar had higher (*p* < 0.05) quantities of OC, N, P, K, EC, and significant pH levels when compared to the soil (Tables 2 and 3). Soils with low phosphorus (P), a medium CEC, and a high sand content at low pH contribute to low fertility (Tables 2 and 3). The low pH of 4.9 indicates the presence of exchangeable aluminum, which can inhibit root development and negatively affect the availability of other nutrients as well as the mineralization of organic matter. Additionally, the very low values of calcium (Ca) and potassium (K) suggest that the soil is highly weathered, resulting in low base saturation. This condition leads to cation exchange sites being occupied by hydrogen ions, which supports the findings of Ref. [24].

Comparing the biochar with the soil, the CH and SB had 118.8 and 128.8 times more OC, 19.7 and 10.2 times more N, 44.2 and 78.3 times more P, and 1.0 and 2.9 times more K than in the soil (*p* < 0.05). However, the EC of the CH was 1.2 times less than in the soil compared to 5.8 times more EC than in the soil (*p* < 0.05). Additionally, CH and SB had 1.9 and 2.1 times more enhancing the pH than in the soil, respectively (Tables 2 and 3). These large differences can contribute to increasing the fertility of the soil. Glaser, Lehmann, and Zech [25] affirmed that the fertilization potential of the biochar is high, especially in tropical soils. This affirmation has been proven by Laird et al. [26] who observed a significant increase in OC, N, P, K, Ca, and Mn after 500 days of biochar addition.

### 3.2. Biochar Effect on Soil Physical Characteristics

#### 3.2.1. Bulk Density

**Figure 3:**
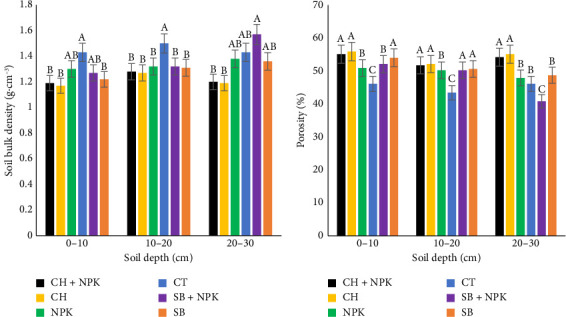
Effects of biochars on (a) soil bulk density and (b) porosity. CH, coconut husk; CH + NPK: coconut husk and NPK; CT, control; SB, sugarcane bagasse; SB + NPK, sugarcane bagasse and NPK. Different letters within a column represent the significant differences at the 5% level of the Duncan multiple range test.

The high bulk density of sandy soil can be attributed to the soil particles. For the soil, bulk density and porosity are inversely (*p* < 0.05) proportional. There were some significant effects (*p* < 0.05) of CH and SB on the soil bulk density (Figure 3(a)). The low bulk density of CH and SB biochar effects on the soil bulk density explained by Laird et al. [26]. There was a significant reduction in the soil bulk density by 19.83% and 11.53%, respectively, in CH and SB compared to CT at 0–30 cm soil depth. In comparison, there was a significant reduction of 7.44% in the bulk density (0–30 cm soil depth) in the CH with bigger biochar particle size compared to SB with smaller biochar particle size on the soil. Smaller biochar particle sizes can decrease the soil bulk density as a results of the arrangement of the biochar particles in the soil void spaces. Since smaller biochar particle size can occupy the pore spaces more effectively as compared to bigger biochar particle sizes. This confirms the findings from Duarte et al. [27]. The reduction in the bulk density is vital for increasing soil porosity, as it directly contributes to root elongation and consequently enhances plant development and production [28]. However, the effects of biochar application under field conditions may not be immediately apparent, as the properties of biochar change over a relatively long time period due to its recalcitrant nature and resistance to decomposition [29].

#### 3.2.2. Porosity

There were significant effects (*p* < 0.05) of biochar application on soil porosity (Figure 3(b)). CH and SB resulted in 20.29% and 13.05% increase in total porosity, respectively, compared to the CT in 0–30 cm soil depth. However, there was 55.47% increase in CH compared to SB in 0–30 cm soil depth. The addition of biochar and consequently increase in the void volume can influence soil physical and hydraulic properties. As bigger biochar particles sizes can occupy more soil void spaces enhancing water to move freely, compared to smaller particles that retain water [30]. Therefore, the addition of biochar improves aeration and water movement resulting in the increase in the total porosity. Numerous studies indicate that the high internal porosity of biochars could support in decreasing bulk density but increasing porosity with ultimate improvement in the water retention capacity of soil [31–35].

### 3.3. Biochar Effects on Chemical Characteristics

#### 3.3.1. Soil pH

**Figure 4:**
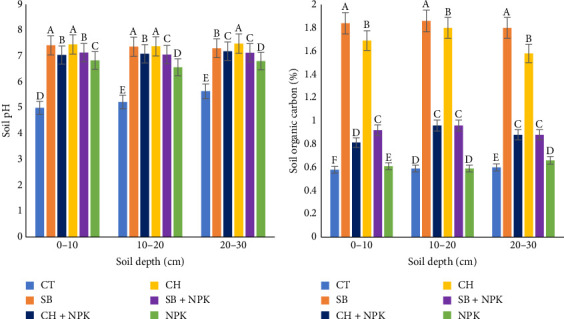
Effects of biochars on (a) pH and (b) organic carbon. CH, coconut husk; CH + NPK: coconut husk and NPK; CT, control; SB, sugarcane bagasse; SB + NPK, sugarcane bagasse and NPK. Different letters within a column represent the significant differences at the 5% level of the Duncan multiple range test.

The application of biochar, both CH and SB, significantly (*p* < 0.05) increased the soil pH across all the soil depths tested (0–10 cm, 10–20 cm, and 20–30 cm; Figure 4(a)) by about 50%. This can be attributed to the inherent properties of biochar that help increase soil pH (Figure 4(a)). During the pyrolysis process, the organic matter in the feedstock is converted into a carbon-rich material with various inorganic compounds, including alkaline cations such as potassium (K), calcium (Ca), magnesium (Mg), and sodium (Na) oxides, carbonates, and hydroxides, which helps increase soil pH [36]. The addition of biochar to the soil releases these alkaline compounds, which could dissolve and interact with the soil solution, thereby increasing the soil pH [37, 38]. The increase in soil pH is directly proportional (*p* < 0.05) to the amount of biochar applied, as more biochar means a higher concentration of these alkaline substances. Furthermore, the increase in soil pH is more pronounced in acidic soils, as the biochar's alkaline compounds can effectively neutralize the acidity.

#### 3.3.2. Soil Organic Carbon (OC)

The application of CH and SB biochar significantly (*p* < 0.05) contributed to an increase of approximately 166.7%–216.7% in OC, respectively, across all soil depths tested (0–10 cm, 10–20 cm, and 20–30 cm (Figure 4(b)). Biochar is a carbon-rich material that can increase the soil OC content due to its resistance to decomposition. Its ability to persist in the soil for extended periods enables long-term carbon sequestration [39]. In addition, the stable structure of biochar can inhibit the surface oxidation of organic matter and reduce the mineralization rate of soil OC, thereby increasing the overall soil OC [40]. Biochar provides a favorable habitat for soil microorganisms, which can enhance the turnover and stabilization of OC in the soil [38, 40].

#### 3.3.3. Available Nitrogen

**Figure 5:**
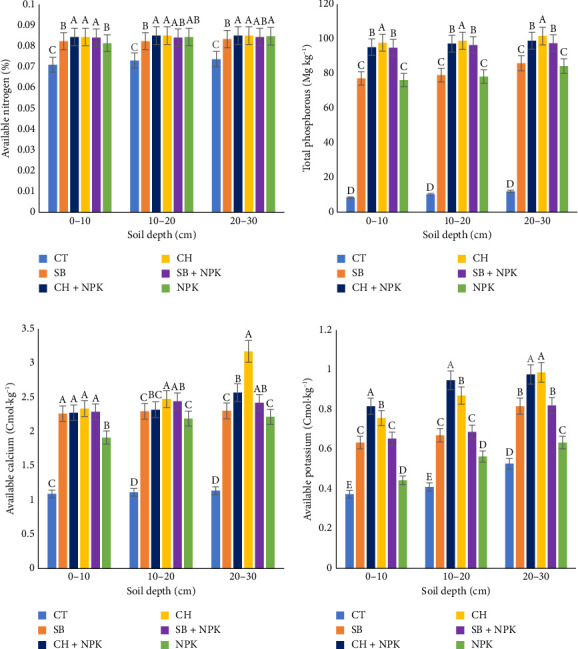
Effects of biochars on (a) available N, (b) total P, (c) available K, and (d) available calcium. CH, coconut husk; CH + NPK: coconut husk and NPK; CT, control; SB, sugarcane bagasse; SB + NPK, sugarcane bagasse and NPK. Different letters within a column represent the significant differences at the 5% level of the Duncan multiple range test.

The application of CH and SB significantly (*p* < 0.05) contributed to an increase of approximately 21.43% in available N across all the soil depths tested (0–10 cm, 10–20 cm, and 20–30 cm) (Figure 5(a)). This finding suggests that biochar application enhanced soil nitrogen (N) availability, N cycling, and N use efficiency, resulting in increased N levels throughout the analyzed soil profile. The mechanisms behind the increased N availability with biochar application are complex. Biochar has been shown to have the capacity to adsorb and retain nitrogen, reducing losses through leaching or volatilization [41]. Additionally, it has been shown to stimulate microbial activity, which may have enhanced N cycling processes, such as mineralization and nitrification [42]. Furthermore, this result highlights the potential of biochar as a promising tool for sustainable agriculture and soil management practices. By enhancing nutrient cycling and productivity while minimizing environmental impacts, biochar can play a crucial role in developing more sustainable and resilient agricultural systems [43].

#### 3.3.4. Total Phosphorus

The application of biochar derived from CH and SB resulted in a significant (*p* < 0.05) increase in total P levels at the 0–10 cm, 10–20 cm, and 20–30 cm soil depths (Figure 5(b)). Interestingly, the increase (24.36%) in total P observed with the coconut-derived biochar was higher (*p* < 0.05) than the increase recorded from the SB biochar (Figure 5(b)). The variations in phosphorus (P) availability between the two biochar types can be attributed to multiple factors. The capacity of biochar to adsorb and retain P is influenced by its physical and chemical characteristics, such as surface area, pore structure, and mineral composition [44]. CH biochar has been shown to have a higher surface area and greater phosphate adsorption capacity compared to SB biochar [45]. Additionally, the mineral composition of the biochar, particularly the presence of calcium, magnesium, and iron, can affect P availability through the formation of stable phosphate complexes [42]. Thus, the enhanced P availability with biochar application is likely due to a combination of mechanisms, including increased adsorption, reduced leaching losses, and improved soil physical and chemical properties that promoted P cycling and plant uptake [43]. These findings also highlight the potential of biochar as a valuable soil amendment for improving P management and enhancing nutrient availability in agricultural systems.

#### 3.3.5. Available Calcium

The application of biochar derived from both CH and SB feedstocks resulted in a significant (*p* < 0.05) enhancement of Ca availability across all the soil depths tested (Figure 5). Specifically, at the 0–10 cm and 10–20 cm depths, the biochar from these two organic sources contributed to an increase of approximately 87.5%–100% of the total Ca in the soil. Furthermore, at the 20–30-cm soil horizon, CH contributed to an impressive 157.6% increase in Ca compared to the CT (Figure 5(c)). The differences in Ca availability between the two biochar types can be attributed to their distinct physical and chemical characteristics. Through mechanisms involving microbial activity, cation exchange, pH modulation, and chemical interactions, biochar effectively improves Ca retention and accessibility. For instance, microbial activity facilitated by biochar contributes to the breakdown of organic matter and the release of bound calcium [46, 47]. Additionally, biochar possesses a high CEC, which may have attracted and retained positively charged ions such as Ca [48]. This characteristic modulated Ca leaching and enhanced its retention within the soil profile [49]. Furthermore, biochar can alter soil pH, creating conditions conducive to improved Ca solubility. The alkaline nature of certain biochars, such as those derived from CH, can raise soil pH levels, facilitating the dissolution of Ca compounds [50], thereby making them more available.

#### 3.3.6. Available Potassium

The application of biochar derived from these two organic feedstock types also showed significant (*p* < 0.05) enhancements in K availability across all the soil depths analyzed (Figure 5(d)). Specifically, SB contributed to an increase of approximately 58.75% of available K at depths of 0–10 cm and 10–20 cm, while achieving 100% availability at the 20–30-cm horizon. In the case of the CH, the available K increased as the soil profile depth increased. At the 0–10 cm, 10–20 cm, and 20–30 cm depths, the CH contributed approximately 95%, 110%, and 150% of the available K, respectively (Figure 5(d)). The observed increase in K availability following the application of biochar derived from organic feedstocks can be attributed to several underlying mechanisms. First, biochar serves as a reservoir for K, effectively retaining and releasing this essential nutrient over time. The high surface area and porous structure of biochar facilitate the adsorption of K ions, preventing their leaching from the soil profile and making them more available [51]. Second, biochar-amended soils often exhibit improved CEC, which enhances the retention and exchange of positively charged ions such as K. This phenomenon leads to increased K availability within the soil matrix. Furthermore, the presence of biochar can influence soil pH, creating conditions conducive to K solubility and mobility [52]. Certain biochar, particularly those derived from alkaline feedstocks like CH, may raise soil pH levels, promoting the release of K ions bound to mineral surfaces and organic matter [43]. Moreover, the application of biochar can stimulate microbial activity in the soil, leading to the mineralization of organic matter [53] and the subsequent release of K. Microbes play a vital role in decomposing organic materials, liberating nutrients such as K for plant uptake [54].

#### 3.3.7. CEC and Electrical Conductivity (EC)

**Figure 6:**
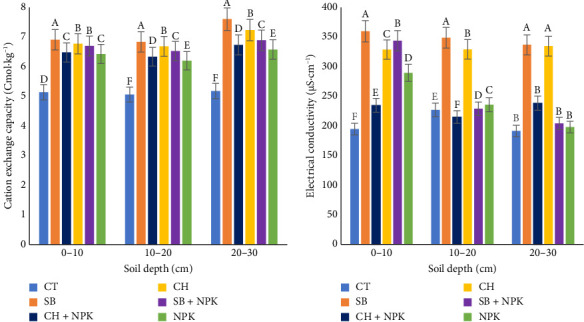
Effects of biochars on (a) cation exchange capacity and (b) electrical conductivity. CH, coconut husk; CH + NPK: coconut husk and NPK; CT, control; SB, sugarcane bagasse; SB + NPK, sugarcane bagasse and NPK. Different letters within a column represent the significant differences at the 5% level of the Duncan multiple range test.

The application of biochar led to a notable increase (*p* < 0.05) in the CEC across soil depths of 0–10 cm, 10–20 cm, and 20–30 cm (Figure 6(a)). Biochar is a medium with a high surface area and porous structure, which provides sites for cation adsorption, enhancing the soil's overall CEC. This increased CEC allowed for greater retention and exchange of positively charged ions [55], including essential nutrients such as K, Ca, and Mg as observed in this study. Additionally, biochar's ability to improve the soil structure and aggregation further contributed to the increase in the CEC, as biochar-amended soils often exhibit improved physical properties such as increased porosity and water retention capacity, creating favorable conditions for cation retention and exchange [56]. Similarly, the application of biochar increased the EC (Figure 6(b)) at the 0–10 cm, 10–20 cm, and 20–30 cm soil depth levels. This could be attributed to the fact that biochar contains various ions and minerals that can contribute to the overall conductivity of the soil solution [57]. When biochar is added to the soil, it releases these ions, increasing the concentration of dissolved salts and, thus, the EC.

### 3.4. Relationships Among Variables, Biplot Analysis, and Hierarchical Cluster Analysis

**Figure 7:**
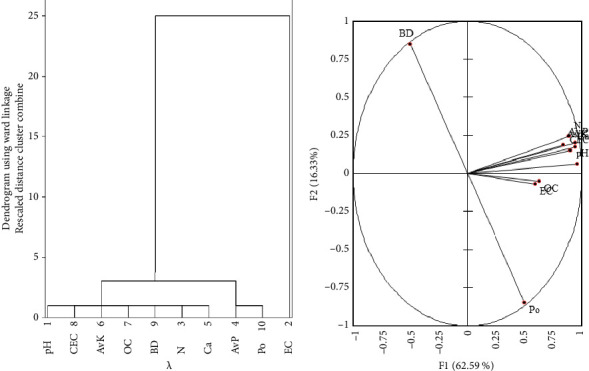
(a): Dendrogram of the ward linkage from the hierarchical cluster, and (b): biplot analysis of soil physicochemical characteristics. Av K, available potassium; Av P, available phosphorus; BD, bulk density; Ca, calcium; CEC, cation exchange capacity; EC, electrical conductivity; N, nitrogen; OC, organic carbon; and Po, porosity.

Correlation analysis was conducted on soil physical and chemical characteristics to show the correlational effects of the biochar application on the soil characteristics (Table 4). The results showed significant (*p* < 0.05) correlations between the parameters, with the strongest correlation (*p* < 0.05) observed between available P and N, and the weakest between porosity and bulk density. The biplot analysis of soil physical and chemical characteristics revealed that the models of accuracy, F1 (62.59%) and F2 (16.33%), collectively explained 78.92% of the experimental variance (Figure 7(b)). In the hierarchical cluster analysis (Figure 7(a)), soil physical and chemical characteristics were grouped based on their roles in property transformation. The analysis yielded two main clusters. Cluster one consisted of two subclusters: one subcluster included pH, CEC, available K, OC, bulk density, N, and Ca, and the other subcluster included available P and porosity. The second cluster consisted of only EC, which showed a link between the soil physical and chemical characteristics. There was a strong correlation among pH, OC, AN, AP, TP, AC, EC, and CEC compared to BD and Po (Figure 7(b)). Similarly, there was a strong correlation among the soil physical and chemical characteristics in Subcluster 1 and Subcluster 2.

## 4. Conclusion

The results of this study showed that biochar application has the potential to improve the physical and chemical characteristics of the soil. The addition of biochar reduced the soil bulk density compared to the presoil condition of the tested soil by significantly improving the porosity. Biochar has great potential for C sequestration, soil fertility enrichment, and reducing the soil pH level from acidic to neutral, which may lead to the improvement in microbial activities. Our study showed that biochar can be recommended for farmers as a soil amendment material to enrich physicochemical characteristics of *Ferric Acrisol* soils. In addition, our study suggests that improved management of biological waste may increase the availability of biochar. Further investigations are recommended to better understand the impact of biochar particle size, application rates, and long-term interactions, as well as the cost-to-benefit ratios on *Ferric Acrisol* (sandy-clay-loam) soils.

## Figures and Tables

**Table 1:** Climatic condition of the experimental site.

Climatic condition	
Coordinate	5°70′20.4 N and 0°19′69.4 W
Rainfall (mm), min (mean) max	1012.53 (1150.48) 1288.42
Temperature (°C), min (mean) max	28 (30.5) 33
Relative humidity (%), min (mean) max	70 (85) 100
Sunshine hours, min (mean) max	138 (190) 242
Soil type	*Ferric Acrisol* (sandy-clay-loam)

**Table 2:** Presoil characterization of the experimental site at 0–30 cm soil depth.

Physical characteristics	
Sand (%)	56.91
Clay (%)	13.56
Silt (%)	29.53
Bulk density (g**·**cm^−3^)	1.5
Porosity (%)	47

**Chemical characteristic**	

pH	4.93
Organic carbon (%)	0.57
Available nitrogen (%)	0.06
Total phosphorus (Mg**·**kg^−1^)	11.07
Available potassium (Cmol**·**kg^−1^)	0.58
Available calcium (Cmol**·**kg^−1^)	1.89
Electrical conductivity (μS**·**cm^−1^)	121.00
Cation exchange capacity (Cmol**·**kg^−1^)	5.34

**Table 3:** Chemical characterization of biochar types.

	CH	SB
pH	9.40	10.24
Organic carbon (%)	67.74	73.42
Available nitrogen (%)	1.18	0.61
Total phosphorus (Mg·kg^−1^)	489.55	866.87
Available potassium (Cmol·kg^−1^)	1.71	1.68
Available calcium (Cmol·kg^−1^)	2.11	1.98
Electrical conductivity (μS·cm^−1^)	100.81	700.20

Abbreviations: CH; coconut husk, SB; sugarcane bagasse.

**Table 4:** Means of Pearson's correlation coefficient among soil physical and chemical characteristics.

	pH	EC	N	Av P	Ca	Av K	OC	CEC	BD	Po
pH	1									
EC	0.602⁣^∗∗^	1								
N	0.870⁣^∗∗^	0.424⁣^∗∗^	1							
Av P	0.912⁣^∗∗^	0.539⁣^∗∗^	0.966⁣^∗∗^	1						
Ca	0.871⁣^∗∗^	0.473⁣^∗∗^	0.888⁣^∗∗^	0.928⁣^∗∗^	1					
Av K	0.795⁣^∗∗^	0.534⁣^∗∗^	0.719⁣^∗∗^	0.774⁣^∗∗^	0.815⁣^∗∗^	1				
OC	0.603⁣^∗∗^	0.255	0.360⁣^∗∗^	0.445⁣^∗∗^	0.546⁣^∗∗^	0.486⁣^∗∗^	1			
CEC	0.852⁣^∗∗^	0.347⁣^∗^	0.828⁣^∗∗^	0.869⁣^∗∗^	0.875⁣^∗∗^	0.691⁣^∗∗^	0.697⁣^∗∗^	1		
BD	−0.425⁣^∗∗^	−0.298⁣^∗^	−0.267	−0.316⁣^∗^	−0.336⁣^∗^	−0.262	−0.330⁣^∗^	0.321⁣^∗^	1	
Po	0.416⁣^∗∗^	0.307⁣^∗^	0.274⁣^∗^	0.322⁣^∗^	0.340⁣^∗^	0.259	0.294⁣^∗^	0.321⁣^∗^	−0.957⁣^∗∗^	1

*Note:* Av K; available potassium.

Abbreviations: Av P; available phosphorus, BD; bulk density, Ca; calcium, CEC; cation exchange capacity, EC; electrical conductivity, N; nitrogen, OC; organic carbon, Po; porosity.

⁣^∗^Correlation is significant at the 0.05 level.

⁣^∗∗^Correlation is significant at the 0.01 level.

## Data Availability

The data that support the findings of this study are available from the corresponding author upon reasonable request.
